# Intelligent Design of Nano-Scale Molecular Imaging Agents

**DOI:** 10.3390/ijms131216986

**Published:** 2012-12-12

**Authors:** Sung Bae Kim, Mitsuru Hattori, Takeaki Ozawa

**Affiliations:** 1 Research Institute for Environmental Management Technology, National Institute of Advanced Industrial Science and Technology (AIST), 16-1 Onogawa, Tsukuba 305-8569, Japan; 2 Department of Chemistry, School of Science, The University of Tokyo, 7-3-1 Hongo, Bunkyo-ku, Tokyo 113-0033, Japan; E-Mails: mitsuru@chem.s.u-tokyo.ac.jp (M.H.); ozawa@chem.s.u-tokyo.ac.jp (T.O.)

**Keywords:** imaging agent, molecular probe, optical signature, smart design, bioluminescence

## Abstract

Visual representation and quantification of biological processes at the cellular and subcellular levels within living subjects are gaining great interest in life science to address frontier issues in pathology and physiology. As intact living subjects do not emit any optical signature, visual representation usually exploits nano-scale imaging agents as the source of image contrast. Many imaging agents have been developed for this purpose, some of which exert nonspecific, passive, and physical interaction with a target. Current research interest in molecular imaging has mainly shifted to fabrication of smartly integrated, specific, and versatile agents that emit fluorescence or luminescence as an optical readout. These agents include luminescent quantum dots (QDs), biofunctional antibodies, and multifunctional nanoparticles. Furthermore, genetically encoded nano-imaging agents embedding fluorescent proteins or luciferases are now gaining popularity. These agents are generated by integrative design of the components, such as luciferase, flexible linker, and receptor to exert a specific on–off switching in the complex context of living subjects. In the present review, we provide an overview of the basic concepts, smart design, and practical contribution of recent nano-scale imaging agents, especially with respect to genetically encoded imaging agents.

## 1. General View of Integrated Nano-Imaging Agents

“Convergence” has been a pivotal strategy of molecular science, e.g., nanobiotechnology for molecular imaging, not only for smart phones. While creation of attractive optical nano-materials emitting fluorescence or luminescence is of fundamental significance, intelligent engineering of the materials practically elucidates their real value. Upon reviewing recent pivotal advances in nanobiotechnology for molecular imaging, integration of multiple functions of nano-materials is witnessed to be a fundamental strategy to address frontier issues of molecular imaging. For instance, therapeutic strategies such as nucleic acid delivery, chemotherapy, hyperthermia, and radiation therapy are combined with one or more imaging functionalities for both *in vitro* and *in vivo* studies [1].

**Figure 1:**
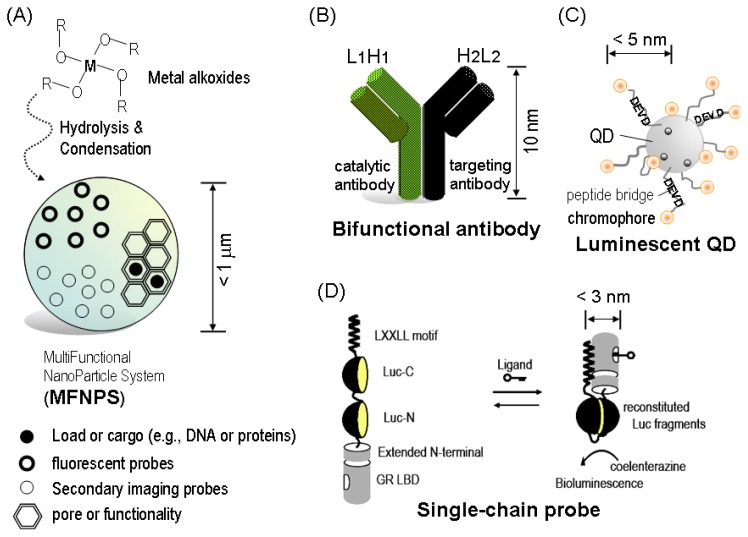
Convergence in construction of nano-imaging agents for molecular imaging in living subjects. (**A**) Schematic illustration of a multifunctional nanoparticle systems (MFNPS). Metal alkoxides undergo hydrolysis and condensation reaction steps to form a metal oxide matrix; (**B**) Bispecific antibody hybrid originating from a corresponding catalytic and targeting antibody; (**C**) Schematic representation of quantum dots (QD) FRET-based protease sensor. Bioluminescence resonance energy transfer (BRET) between the chromophore and QD is quenched by a cleavage of active caspase-3 to the specific substrate bridge, Asp-Glu-Val-Asp (DEVD); (**D**) Schematic diagram of the working mechanism of a single-chain imaging probe. A luciferase (Luc) is fragmented into two parts, which are sandwiched between the ligand binding domain of glucocorticoid receptor (GR LBD) and an LXXLL motif. Activated GR LBD binds to the LXXLL motif. This exerts reconstitution of the fragmented luciferase.

Elaboration of multifunctional nanoparticle systems (MFNPS) is a good example of this view (Figure 1A). MFNPS is synthesized through summarization of individual, functional ingredients, *i.e.*, fluorophore, magnetic or radio-active material, porous structure, and biologically active cargo. Thus, MFNPS is capable of exerting fluorescence and magnetic resonance imaging (MRI) as optical signatures and can carry drugs, nucleic acids, or other functional proteins to disease area [2].

Catalytic antibodies may be another prototypical example of the idea of convergence. Antibodies may be the most popular tool for molecular imaging and recognition science. The concept of catalytic antibody was developed and utilized for chemotherapy and molecular imaging missions, where the antibody is capable of catalyzing a chemical reaction, similar to enzymes. Furthermore, this catalytic antibody was hybridized with other classical antibodies, and was named “*bifunctional antibody*” because the antibody exerts bispecific properties onto the targets [3] (Figure 1B). These multifunctional antibodies were then labeled with a variant of *Renilla* luciferase (RLuc) for cancer imaging [4]. This basic strategy was further expanded to a concept of “*chemical adaptor*”, which combined a tumor targeting moiety, an enzyme substrate, and a drug into a singular entity [5].

Luminescent quantum dots (QDs) are another example of this idea of convergence. QDs themselves are simply inorganic semiconductor nanoparticles of 1–10 nm in diameter, which size-dependently emit visible colors [6,7]. These luminescent QDs can be coated with a specific peptide-bridged chromophore. This integration exerts a fluorescence resonance energy transfer (FRET) between the QD and chromophore, the peptide bridge of which is digested by a specific protease [8] (Figure 1C).

Single-chained fusion protein probes also exhibit a good example of this idea of convergence. In this kind of molecular probes, the *N*- and *C*-terminal fragments of split-luciferase, a signal recognition protein, and appropriate linkers are integrated into a single molecular backbone (Figure 1D). In the presence of a ligand, the signal recognition protein dramatically transforms and the adjacent *N*- and *C*-terminal fragments of split-luciferase are finally reconstituted to emit bioluminescence. This resurrected bioluminescence reflects activities of the ligand in a host organism. This type of probe is called “*single-chain probe*” or “*integrated-molecule-format (IMF) probes*”, because all of the components for ligand-sensing and bioluminescence emission are integrated into a single chain [9,10]. This basic technology has been further extended to multicolor imaging of bifacial effects of a ligand [11]. Multiple single-chain probes emitting various colors have been introduced into an organism to simultaneously image multifacial effects of a ligand.

“Smart integration” is a key feature for creating excellent molecular imaging agents. Upon integrating several exogenous components in a single entity, sophisticated adjustment of linkers and optimization of dissection sites of each component become fundamental issues. The linkers in nano-imaging agents take up multiple roles, including an artificial hinge, enzyme substrate, and/or spacer. These linkers can be made of functional peptides, chemically active functional groups, or polymers, according to the character of the imaging agents. Peptide linkers have been well reviewed previously [12]. Through these integrated designs, the optical probes indeed act as a smart molecular imaging probe in the host organism.

## 2. Organic and Inorganic Nano-Imaging Agents

Designing nano-imaging probes is crucial to the success of a molecular imaging study. Well-designed molecular probes can reveal spatiotemporal information on a specific molecular biological process in living subjects [13].

To date, organic and inorganic nano-imaging agents have been broadly utilized for addressing a variety of frontier molecular imaging missions. Meanwhile, genetically encoded imaging agents are emerging and are rapidly substituting the realm of conventional organic and inorganic imaging agents.

### 2.1. Synthetic Organic Agents

Conventional molecular imaging technologies for ion imaging have largely depended on synthetic organic probes. The pivotal strategy for designing organic probes is that a known fluorophore, such as fluorescein or rhodamine, is chemically modified with a known metal ion receptor. These organic probes enable to trace changes in intracellular metal ion concentrations and distributions, as well as mobilization of metal ion pools upon physiological or pathological stimulation. New efficient chemical design of the probes may offer the ability to perform new types of molecular imaging experiments that are currently unavailable.

For instance, a rhodamine derivative, RhodZin-3, was previously studied for tracing mitochondrial Zn^2+^ homeostasis [14] and in studies of the functions of the zinc transporter, Znt5B [15]. FuraZin and IndoZin are Zn^2+^ sensors derived from the classic Fura and Indo Ca^2+^ probes and operate via an internal charge transfer mechanism, in which metal ion binding to the probe modulates the electron-donating properties of the electron-rich receptor [16]. Fluorescein-based Zn^2+^ probes were also developed and named as ZnAF series indicators. In addition to fluorescein- and rhodamine-based indicators, cell-compatible Zn^2+^ sensors have been developed using the backbone of BODIPY [17], naphthalimide [18], cyanine [19], and coumarin [20] dyes.

Furthermore, as an example for peptide-sensing, Griffin *et al.* developed a synthetic chemical indicator that can recognize six natural amino acids (cysteines) that may be genetically incorporated into proteins of interest. The fluorescein-derived indicator, named “FLAsH”, recognizes four cysteines placed at the *i*, *i* + 1, *i* + 4, and *i* + 5 positions of a α-helical peptide [21,22].

### 2.2. Inorganic Nano-Imaging Agents

As an inorganic nano-imaging agent, QDs are representative for molecular imaging. QDs are semiconductor nanoparticles of 1–10 nm in diameter. These luminescent nanocrystals are composed of atoms from the II–VI (CdS, CdSe, CdTe, ZnO, ZnSe), III–V (InP, InAs, GaN, GaP, GaAs), and IV–VI (PbS, PbSe, PbTe) groups of the periodic table [23]. CdX QDs (X = S, Se, Te) are especially attractive because they emit electromagnetic spectrum in the range of ultraviolet, visible, and near-infrared. The emission color of the QDs is size-dependent [6,7].

However, cytotoxicity is a big issue for QDs. Class A element (Cd, Pb, and Hg) based QDs show cytotoxicity *in vitro* under extreme conditions [24]. To avoid this problem, these elements can be replaced by other more benign elements, such as CuInS_2_/ZnS core/shell QDs.

Near-infrared light (700–900 nm) efficiently penetrates skin and blood more than visible light because these tissues scatter and absorb less light and low autofluorescence at longer wavelengths [25]. Near-infrared light also has potentially high spatial resolution, sensitivity, and low risk to living subjects because it utilizes non-ionizing radiation [24]. Therefore, the desirable optical property of nano-imaging agents for tissue imaging is emission of near-infrared light.

A smart design of nano-imaging agents emitting near-infrared light was reported with name of “*self-illuminating nanoprobes*” [26,27]. The surface of QDs was covalently coupled to a donor of bioluminescence resonance energy transfer (BRET), RLuc8. Energy from a chemical reaction catalyzed by the donor luciferase is transferred to the linked QD emitting near-infrared light. This molecular design has enabled us to carry out self-illuminative imaging of cancers in the deep tissue of living subjects without the need for external excitation.

When compared with organic nano-agents, QDs possess higher photostability and brightness, but exhibit toxicity and poor *in vivo* biodistribution. Rational design of new QD imaging agents is deeply related to the surface labeling chemistry. The general principle for QD labeling is that first the QDs are made water-dispersible and then are bound to biomolecules. This labeling can be achieved electrostatically, via biotin–avidin interactions, by covalent cross-linking or by binding to polyhistidine tags [28].

As organic and inorganic nano-scale imaging agents have been well reviewed in previous papers [1,13,24], we have focused on genetically encoded nano-imaging agents.

## 3. Genetically Encoded Nano-Imaging Agents

### 3.1. Overview on the Construction of Fluorescent Probes

Since green fluorescent protein (GFP) was first extracted from jellyfish *Aequorea victoria* by Dr. Shimomura [29], fluorescent proteins have been popularly utilized in a variety of molecular imaging studies.

This native fluorescent protein (FP) was then carefully modified with mutagenesis to improve its optical properties, such as red color-emitting spectra, fast folding properties, enhanced fluorescence intensity, low sensitivity to ions, and temperature stability. A crystallographic study on the structure of GFP revealed regular β-barrels with 11 strands on the outside of the cylinder. These cylinders were found to have a diameter of about 3 nm and a length of about 4 nm [30]. In addition to wild-type GFP, the discovery of novel GFP-like proteins from Anthozoans (coral animals) has significantly extended the range of colors from green to red.

The characteristic of FP-based probes is that they smartly make use of the optical properties of FPs. For instance, a photoconvertible fluorescent protein was first developed by Dickson *et al.* in the process of GFP mutation [31]. Since then, FPs with changing fluorescence intensity (photoactivation) or color (photoconversion or photoswitching) in response to light of a specific wavelength have been discovered. The photoconversion of FPs, such as Kaede, has been explained as the break or extension of π-conjugation in the chromophore of Kaede in response to specific light [32]. Similarly, illumination of an FP named Dronpa reversibly switches on and off by emission at 488 and 405 nm. These interesting optical characters of some FPs imply that they can act as excellent fluorescent probes even without any artificial modification to the FPs. In fact, in the early days’ design of probes, GFP was simply fused to a protein of interest.

The basic designs of fluorescent probes have been further elaborated to create more complex imaging probes, which include probes with circular permutation (CP) and FRET. CP is an interesting design for fabricating nano-imaging probes embedding GFP variants, where the β-barrels of GFP are rearranged. To exert an on–off switch to the structure of GFP, the original *N*- and *C*-terminals of GFP have been fused with a short peptide linker, whereas a new *N*- and *C*-terminals have been created at the hinges of the β-barrels. Subsequently, the new terminals have been extended with a pair of proteins of interest, respectively. This artificial crack to the cylindrical structure results in disruption of fluorescence because of solvent penetration to the hydrophobic chromophore within the cylinder. The fluorescence is resurrected by the closure of the crack through the binding of the protein pair [33,34].

Many of the fluorescent probes based on protein-fragment complementation and reconstitution are unfortunately irreversible and time-consuming for chromophore formation. This irreversible feature destroys the quantitative fidelity of the results and limits the analysis of temporal interactions of proteins of interest.

Majority of FP-based imaging probes make use of FRET, which is an excellent platform for imaging the molecular dynamics of proteins in cells. The FRET-based probes make use of the ability of a higher energy donor fluorophore to transfer energy directly to a lower energy acceptor. The energy donor and acceptor fluorophores are fused to a pair of proteins of interest. Upon interaction between the pair of proteins in the probe, the relative distance between the donor and acceptor fluorophores varies. On the other hand, upon dissociation of the pair of proteins, FRET disappears. Therefore, this method is based on reversible, intracellular protein–protein interactions. The working mechanism of FRET-based probes is explained in detail in Section 4.1.

### 3.2. Labor-Effective Fabrication of Bioluminescent Probes

Beetle and marine luciferases are attractive imaging agents for bioanalysis and molecular imaging. The light-emitting reaction is very simple, harmless, and does not require an external light source or cofactors [35]. Unlike other organic fluorescent agents or inorganic nanoparticles, which have to be delivered to the imaging target, bioluminescent probes can be genetically encoded and expressed *in situ*. The bioluminescent probes do not need external light source for luminescence and thus background intensities can be significantly reduced [13].

Recent engineering of luciferases with designed properties and functionalities represents an important direction for molecular imaging and bioassays. Many of the native luciferases are unstable and poor in optical intensity. These native luciferases have been modified through a random and site-directed mutagenesis for enhancing the optical intensities and prolonged emission half-life [36].

Random mutagenetic approach of luciferase engineering is normally slow and tedious, and enforces severe consumption of time and labor. In particular, in the absence of crystallographic data, no appropriate measures are available for luciferase engineering. Thus, several rational strategies to manipulate luciferases have been developed to relieve the efforts on luciferase engineering.

A *semi-rational* strategy for luciferase engineering, called “*a consensus sequence-driven mutagenesis strategy*”, was demonstrated in a study by Steipe *et al.*[37]. An alignment of relative protein sequences is known to reveal frequently occurring and less occurring amino acids. It is considered that frequently occurring amino acids at a given position have a larger thermostabilizing effect than the less frequent amino acids [38]. In this sense, mutation sites and kinds of amino acids are decided accordingly to increase the frequently occurring amino acids in the alignment.

**Figure 2:**
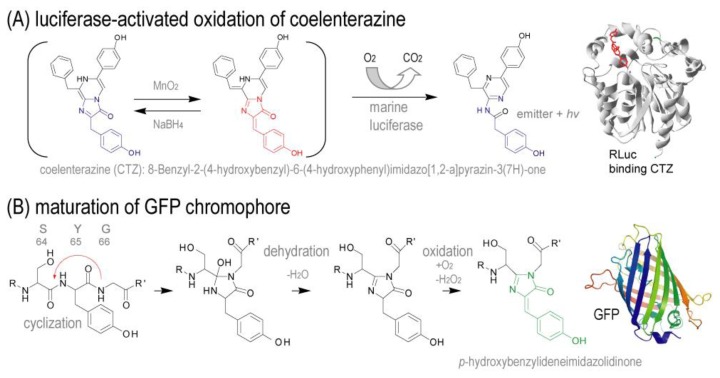
Lighting mechanisms of marine luciferases and fluorescent proteins. (**A**) Marine luciferases oxidize coelenterazine (CTZ) to emit bioluminescence; (**B**) Chromophore of GFP, ^65^SYG^67^, is matured by oxidation. The chemical structural backbone is similar to that of CTZ. Abbreviations: CTZ, coelenterazine; GFP, green fluorescent protein; RLuc, *Renilla* luciferase. This figure was obtained from a reference by Dr. Kim [10].

Kim *et al*. also introduced a *semi-rational* strategy for effective luciferase engineering [10,39]. They interestingly focused on the chemical structural similarity between the fluorophore of GFP and the common substrate of marine luciferases, coelenterazine (Figure 2). Based on this structural comparison, they speculated that GFP variants embed the chromophore inside the molecular backbone, whereas marine luciferases recruit the choromophore (we call it “*substrate*”) from the outside. In this view, they speculated that a *hydrophilic interface* in the luciferases provide a favorable platform for substrate recruitment (we call this as “*active site*”).

**Figure 3:**
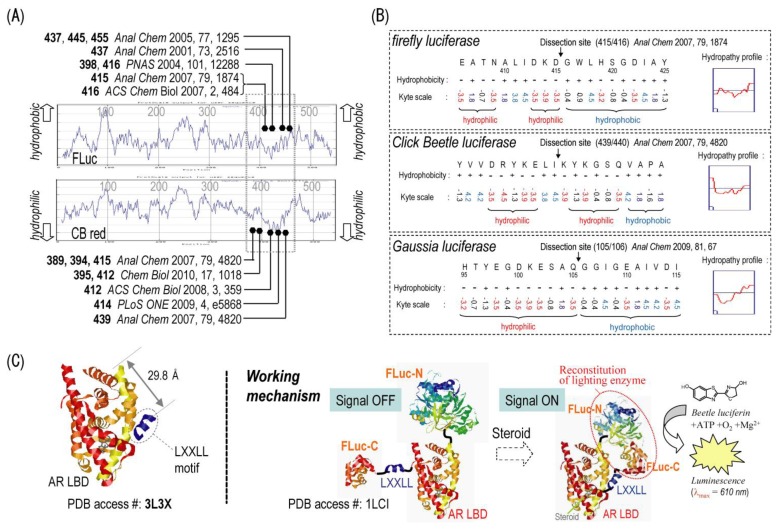
Brief illustration of the overall fabrication process of single-chain probes. (**A**) A hydrophilicity search of FLuc and click beetle luciferase red (CBLuc Red), revealing a significantly hydrophilic region in the middle of the amino acid sequences. The black circles with references indicate specific dissection sites in the sequence. The respective references for dissection sites are: 437 [45], 445 [45], 455 [45], 437 [46], 398 [44], 416 [43,44], 415 [9] for FLuc; 389 [47], 394 [47], 415 [47], 395 [48], 412 [11,48], 414 [49], and 439 [47] for CB Red; (**B**) A highlighted amino acid sequence of CBLuc Red exhibiting the dissection sites. The respective references for dissection sites are: 415/416 for FLuc [9], 439/440 for CBLuc [47], and 105/106 for *Gaussia* luciferase (GLuc) [50]; (**C**) Schematic illustration of an anticipated molecular action of the single-chain probe sensing androgen. In the presence of androgen, the activated AR LBD binds to an LXXLL motif. The binding of AR LBD–LXXLL motif further induces reconstitution of the adjacent split-FLuc.

Accordingly, Kim *et al*. suggested a hydrophilicity search of luciferases to assume a putative active site as an engineering target of the luciferases for mutation and dissection, prior to empirical engineering, because a hydrophilic interface of luciferases should be advantageous to recruit the substrate in the aqueous phase. As shown in Figure 3A, this view is supported by many precedent studies on luciferase-based assays [11,40–44]. This characteristic hydrophilic region is interestingly highly conserved and comprises a drastic interface between highly hydrophilic and hydrophobic amino acids in the sequence of beetle and marine luciferases (Figure 3B). This region has been frequently utilized in the engineering as an optimal dissection or mutation target. The firefly luciferase (FLuc) is fragmented into two parts at amino acid 415, which are sandwiched between the ligand binding domain of glucocorticoid receptor (GR LBD) and an LXXLL motif. In the presence of androgen, the activated AR LBD binds to an LXXLL motif. The binding of AR LBD–LXXLL motif further induces reconstitution of the adjacent split-FLuc and light emission (Figure 3C).

### 3.3 Tutorial Instruction for the Fabrication of Single-Chain Probes

In this section, we list the prototypical procedure for fabricating a single-chain probe with split-FLuc.

- The amino acid sequence of FLuc can be obtained from the public database, the National Center for Biotechnology Information (NCBI; accession #: M15077).
- The hydrophilicity search of the amino acid sequence of FLuc can be conducted in a specific web service for biological information, e.g., Swiss Institute of Bioinformatics (SIB)’s ExPASy Proteomics service [51]. The scale of *Kyte* and *Doolittle* is recommended.
- The hydrophilicity search should reveal a remarkably hydrophilic region at the 4/5th region from the beginning, in the case of FLuc.
- A series of FLuc fragments is generated through a consecutive dissection of the sequence in the chosen hydrophilic region. This fragmentation exerts a temporal inactivation of FLuc. Upon decision of the fragmentation sites, flexible amino acids such as glycine (G) and alanine (A) are preferred, because they are frequently observed in hinge regions and thus minimize decomposition risk after the expression of the fragments.
- A protein of interest, e.g., the ligand binding domain of nuclear receptors (NR LBD) and protein kinases, are sandwiched between the fragmented FLuc via a flexible glycine/serine (GS) linker. This step generates a series of single-chain probes. Nuclear receptors (NR) are preferred because they exert great structural conversion in the presence of an agonist. Optimization of the linker length is tedious and time-consuming. Thus, we recommend researchers to first optimize the length of NR LBD or FLuc. Furthermore, we recommend carrying out examination of the linker length as the final step.
- The relative optical intensity and ligand sensitivity of the probes is examined with an appropriate assay kit, such as Bright-Glo (Promega). Bioluminescence intensity and stability are heavily influenced by buffer conditions such as pH, additives, and temperature. An optimal buffer set according to luciferases may minimize these fidelity risks in signals.
- According to the initial evaluation results, further optimization on the linker length and dissection sites of FLuc is conducted. Extension of the length of NR LBD may also be effective. Routine design fixation and evaluation shape a better efficient imaging agent.

This basic procedure may be modified according to the characters of luciferases.

When designing a single-chain probe, flexible linker is a difficult issue to examine. Although great efforts have been devoted to create excellent linkers for ideal integration of the components in a single entity, elaboration of an efficient linker still remains a challenging mission. In any case of the linkers, their intended control is practically difficult owing to unexpected features of single-chain probes, such as unpredictable steric hindrance. Therefore, researchers may consider the following practical strategies as a labor-saving choice of developing linker peptides: (i) to mimic the success stories of fusion protein probes that were previously well approved and (ii) to gain cumulative experience based on trial-and-errors.

## 4. Examples of Smartly Designed Molecular Probes

Genetically encoded molecular probes are versatile and their scope is rapidly expanding with the development of new fluorescent and bioluminescent colors, chemistries, and probes [52]. Unlike conventional passive probes, such as luminescent QDs and net reporter proteins that switch “irreversibly-on” or “always-on” luciferase activity enables the design of “smart” probes: (i) that can be temporally switched “on” from an “off” state by a ligand; and (ii) that can be reversibly switched “off” from an “on” state by deprival of the ligand. Such reversibility and versatility promise high signal-to-noise (S/N) ratios and the quantitative nature of the optical signature. With this critical feature of fluorescent proteins and luciferases, several uniquely designed probes have been fabricated and utilized in various physiological samples and living subjects.

All of the genetically encoded probes can be divided into two major families: (i) a family of genetic and transcriptional probes (GTP) and (ii) a family of nontranscriptional probes (NTP). The typical feature of GTP is to accompany *transcription* of a reporter protein such as light-emitting proteins or enzymes in one step of the protocol. However, this family of probes requires a long ligand-stimulation time until sufficient accumulation of the reporter protein is reached. A reporter-gene assay and a two-hybrid assay are based on this family of probes.

On the other hand, NTP are expressed beforehand and prelocalized in adequate intracellular compartments of interest. The optical intensity is ready to be developed upon the host cell being stimulated by a signal.

The NTP are further categorized into the following major groups according to their working mechanisms: (i) FRET; (ii) BRET; (iii) Chemiluminescence Resonance Energy Transfer (CRET); (iv) Protein-Fragment Complementation Assay (PCA); and (v) Intein-Mediated Protein-Splicing Assay (PSA); among others.

### 4.1. FRET

The principle of FRET is based on energy transfer from a higher energy donor fluorophore to a lower energy acceptor fluorophore [53]. The energy donor and acceptor fluorophores are fused to a pair of proteins of interest. Upon interaction between the pair of proteins in the probe, the relative distance between the donor and acceptor fluorophores varies. The variance in the distance exerts a color change. Efficient FRET probes are designed to address the following concerns: (i) optimizing the brightness (quantum yield (QY) and extinction coefficient (ɛ)) between the donor and acceptor proteins; and (ii) minimizing spectral cross-over between the donor and acceptor. For instance, the acceptor can be directly excited with a light source, instead of the donor. This should exhibit false-positive fluorescence.

Molecular imaging with FRET is one of the most popular methods for observing the spatial and temporal dynamics between two distinct proteins of interest in living cells. FRET provides a very sensitive measure of small changes in intermolecular distances. Therefore, the presence of FRET is a good indicator of close proximity, implying protein–protein interactions.

A key strategy to fabricate an efficient FRET probe is to use potential fluorescent protein pairs exerting excellent photophysical properties, such as high extinction coefficient, QY, photostability, and greater Förster radius R_0_.

**Figure 4:**
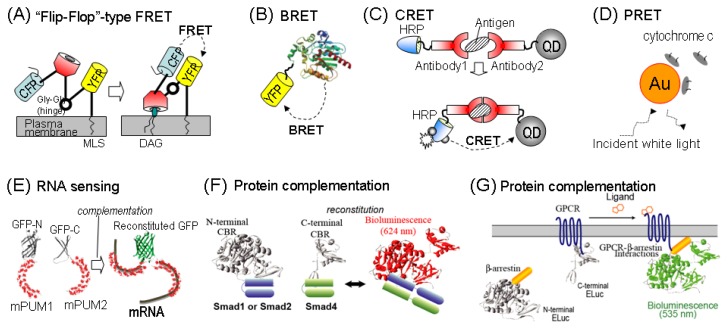

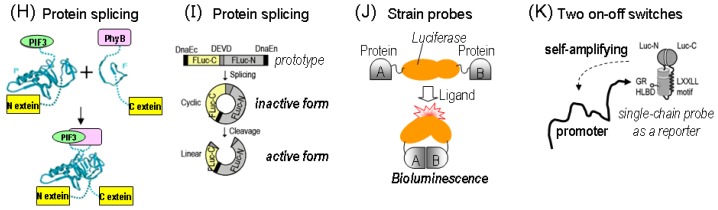
(**A**) A “Flip-Flop”-type indicator (Fllip). This probe is designed to be fixed on the plasma membrane (PM) and dramatically transforms in the presence of a second lipid messenger, PIP_3_; (**B**) A BRET probe called “BAF-Y.” The resonance energy by RLuc8 is transferred to the adjacent fluorescent protein “EYFP”; (**C**) A gold nanoparticle (AuNP) based CRET system. AuNP and HRP are conjugated to two different antibodies. When the two antibodies bind to a same antigen, this immunocomplex quenches CRET between HRP (energy donor) and AuNPs (energy acceptor); (**D**) A probe set based on PRET. The PRET imaging relies on the resonant plasmonic energy transfer from a gold particle to a conjugated target molecule; (**E**) A GFP reconstitution by complementation. PUMILIO is an RNA-binding protein. When mPUM1 and mPUM2 sense the corresponding mRNA sequences, the adjacent *N*- and *C*-terminal fragments of EGFP are complemented; (**F**) A multicolor imaging probe. Multiple luciferases emitting various colors are fragmented in the same probe set, and are conditionally reconstituted, according to which the signal is activated; (**G**) A PM imaging probe. Interaction of G-protein coupled receptors (GPCRs) with β-arrestin on the PM was imaged with a strategy of luciferase complementation; (**H**) A mechanism of light-induced protein splicing. The proteins, PhyB and PIF3, are dimerized by light. The split*-*VMA intein (I^N^ and I^C^) is thus reconstituted. The figure was modified from the reference [56]; (**I**) A mechanism of protein splicing by caspase 3. The expressed precursor of DnaE and FLuc is spliced in a closed circular form. The circular form is linearized by caspase-3; (**J**) A mechanism of molecular strain probes. Intramolecular protein–protein interaction by estrogens exerts a distortion to the full-length luciferase; (**K**) A mechanism of an imaging probe with two on–off switches. In stead of a full-length luciferase, the reporter system carries cDNA encoding a single-chain probe. Glucocorticoid is recognized two times: one is by the expression machinery for a glucocorticoid response element (GRE), and the other is by the expressed single-chain probe, which has a weak self-amplifying ability.

An interesting design of a FRET probe was previously reported with the name of “Flip-Flop”-type indicator or simply “Fllip” [54,55] (Figure 4A). This probe makes use of two GFP variants and their energy transfer is similar to a common FRET probe. The distinctive feature of this probe is the rigid α-helical linkers inserted between the probe components. This probe is designed to be fixed on the plasma membrane (PM) and remain inactive in the basal condition. However, the probe dramatically transforms in the presence of a second lipid messenger, PIP_3_. This intramolecular conformation change varies the relative distance between the incorporated GFP variants, which results in color change.

### 4.2. BRET

BRET is similar to FRET with respect to the resonance energy transfer (RET). In fact, BRET shares a similar working mechanism with FRET, except that the donor molecule is a luciferase, not a fluorescent protein. The resonance energy by a luciferase is transferred to an adjacent fluorophore.

BRET is a useful technique to access protein–protein interactions because the BRET-permissive distance of less than 10 nm is very similar to the dimensions of biological macromolecular protein complexes [57].

An interesting design of BRET probe was demonstrated by Hoshino *et al.*[58] (Figure 4B). The probe named “BRET-based Autoilluminated Fluorescent Protein on EYFP (BAF-Y)” exhibited an extraordinary efficiency in the RET between a variant of *Renilla* lucicferase (RLuc8) and EYFP. This excellent RET was achieved simply through an optimization of the linker length and selection of the luciferase and fluorescent protein.

Although BRET between luciferases and fluorescent proteins is well known, that between luciferases and QDs has also been represented previously. In the probe design, *Cypridina* luciferase (CLuc) and QD were conjugated to biotin and streptabidin, respectively. The biotin–streptavidin reaction resulted in BRET between CLuc and QD [59].

### 4.3. CRET

The above-mentioned bioluminescence is a member of chemiluminescence. Many chemical reactions emitting light have been utilized for bioassays. Although being less familiar than FRET and BRET, CRET has been utilized in the design of various nano-imaging agents [60] (Figure 4C). CRET is a nonradiative RET process, in which the excited donor is produced by a *chemical reaction*. A molecular design of CRET was typically elucidated by conjugation of an energy donor (e.g., horseradish peroxidase (HRP)) and an energy acceptor (e.g., QDs). For example, gold nanoparticle (AuNP) based CRET systems have been reported previously. In the probe design, AuNP and HRP were conjugated to two different antibodies. When the two antibodies bind to a same antigen, it indicates construction of a sandwich immunocomplex. This immunocomplex quenches CRET between HRP (energy donor) and AuNPs (energy acceptor) [61].

### 4.4. PRET

Nanospectroscopy with PRET is another example of advancement in molecular imaging in living cells. PRET imaging relies on the resonant plasmonic energy transfer from a gold nanoplasmic probe to conjugated target molecule.

The imaging mechanism is illustrated in Figure 4D. When fluorescent molecules are close to the surface of a metal nanoparticle, their fluorescence increases, which is called metal-enhanced fluorescence (MEF) where the optimal distance is about 10 nm. Quenching of the fluorescence occurs when the fluorophore is located much closer to the metal surface than that for MEF. PRET suppresses light scattering from nanoparticles when resonance-matched molecules, such as metal complexes or dyes, are in the vicinity of the nanoparticles [62]. The scattering spectrum of PRET creates quantized quenching dips within the Rayleigh scattering spectrum of the probe [63]. The spectrum change in response to the functionalized PRET probe to various environmentally and biologically relevant metal ions appears significant.

### 4.5. Protein-Fragment Complementation Probes

Protein-fragment complementation-based probes (PCP) are one of the major tools for determining protein–protein interactions. The basic design of PCP is as follows: (i) monomeric reporter proteins such as GFP and luciferase are first dissected into *N*- and *C*-terminal fragments for the temporal inactivation of the optical properties; (ii) a set of proteins of interest is linked to each of the fragments of the reporter protein via appropriate linker peptides for unity. Upon interaction between the pair of proteins, the adjacent fragments of the reporter protein are approximated. The restored optical intensity is determined with an appropriate detector. One challenge for the development of complementation strategies is the determination of the optimal dissection sites of luciferase in the PCP. Optimization of the split sites in luciferase are of critical importance for guaranteeing temporal inactivation and conditional reconstitution of the fluorescence or luciferase activity.

Many PCPs have been designed for complementation between two independent fusions. In fact, such analyses are validated on the premise that the two component fusions are expressed equally beforehand. Biased expression of the component fusions inevitably exerts their inefficient complementation. To overcome this limitation, Kim *et al*. demonstrated a series of single-chain bioluminescent probes, where all components for ligand sensing and light emission were integrated into a single backbone. In contrast to the precedent *inter-*molecular complementation, this type of probes provides an *intra*-molecular complementation [64].

The above-mentioned basic design of PCP has been applied in various directions. CP of split-luciferase in a single-chain probe is an interesting design for low S/N ratios [65,66] (Figure 1D). In this probe, the active site in the luciferase is first dissected into two fragments. The *N*- and *C*-terminal fragments are replaced in a *C*- and *N*-terminal order. This means that the active site fragments are placed at the opposite sides of each other. In this molecular design, the encountering chances between the active site fragments dramatically decrease, which consequently minimizes the background intensities.

The concept of PCP was further extended to visualize mRNAs in living cells (Figure 4E). PUMILIO is an RNA-binding protein that comprises an array of RNA-recognizing amino acids. In the unique probe design, two RNA-binding domains of human PUMILIO1 (e.g., mPUM3 and mPUM4) are fused with *N*- and *C*-terminal fragments of split-EGFP, respectively. When the mPUM3 and mPUM4 sense the corresponding mRNA sequences in living cells, the adjacent *N*- and *C*-terminal fragments of EGFP are complemented, resulting in resurrection of strong fluorescence. This strategy was successfully applied to determine mRNA of β-actin [67] and endogenous mitochondrial mRNA [68].

Another tendency in the molecular design of PCPs is to construct a set of multicolor imaging probes, where multiple luciferases emitting various colors are fragmented in the same probe set, and are conditionally reconstituted according to the signal that is activated; e.g., whether the signals are agonistic or antagonistic [11]; or whether Smad4 binds to Smad1 or Smad2 (Figure 4F) [49].

The basic concept of PCPs is also utilized for imaging molecular events on the PM. For instance, interaction of G-protein coupled receptors (GPCRs) with β-arrestin on the PM was imaged with a set of bioluminescent probes [69,70] (Figure 4G). Similarly, dimerization of extracellular signal-regulated kinase 2 (ERK2) was visualized with a pair of ERK2-linked split-RLuc [40].

### 4.6. Intein-Mediated Protein-Splicing Probe

Protein splicing is a naturally occurring, posttranslational processing event involving precise excision of an internal protein segment (*intein*) from a primary translation product, *i.e.*, external protein (*extein*). At intein–extein junctions, conserved amino acid residues are directly involved in the protein-splicing reaction. An important feature of protein splicing is a self-catalyzed excision of the intein and ligation of the flanking exteins without any exogenous cofactor or energy source [71].

This natural occurrence for protein maturation was successfully utilized for designing a new series of molecular probes. In the classic probe design, *N*- and *C*-terminal fragments of split-VDE intein (1–184 amino acids and 389–454 amino acids) were respectively linked to *N*- and *C*-terminal domains of split-GFP (1–128 amino acids and 129–239 amino acids). This pair of fusions was further labeled with calmodulin (CaM) and its target peptide (M13), respectively [72].

Ca^2+^-bound CaM interacts with M13. This interaction provokes a protein splicing reaction between the approximated *N*- and *C*-terminal fragments of VDE, generating a full-length GFP.

Conditional protein splicing was well demonstrated by Tyszkiewicz *et al*., who developed a protein splicing system activated by red (660 nm) and far-red (750 nm) light in yeast [58] (Figure 4H).

### 4.7. Luciferase Cyclisation by Protein Splicing

An innovative design called “*luciferase cyclisation*” among reporter reconstitution technologies was previously reported [73,74] (Figure 4I). In the probe design, the *N*- and *C*-terminal fragments of FLuc were circularly permutated via a substrate peptide of caspase-3 (DEVD). The *N*- and *C*-terminal ends of the CP FLuc were further linked to the *N*- and *C*-terminal fragments of a DnaE intein. After translation into a single polypeptide, the *N*- and *C*-terminal ends were self-catalytically ligated by protein splicing, creating a closed circular fusion protein. As the molecular structure is distorted and strained by a tension, CP FLuc temporarily loses its enzymatic activities (*closed circular form*). In the presence of an apoptosis signal, active caspase-3 digests the DEVD sequence in the circular fusion and linearizes the formation (*opened linear form*). In the linear form, the CP FLuc restores the optical intensities, which indexes activity of caspase-3.

### 4.8. The Other Probe Designs

Unique molecular designs in genetically encoded nano-imaging agents have been developed. A unique bioluminescent probe named “*molecular-tension probe*” was developed on the basis of molecular tension of a luciferase that is artificially appended by protein–protein binding [75] (Figure 4J). Unlike other probe design, this probe makes use of the full length of a luciferase, which is sandwiched between a pair of proteins of interest. In the probe design, the linker length between the ingredients is minimized to exert an efficient molecular tension to the sandwiched luciferase. This simply designed molecular probe is surprisingly sensitive to estrogens.

All of the genetically encoded probes are roughly divided into two categories: GTP and NTP. Recently, a probe system that combines the characteristics of the two groups has been developed [65] (Figure 4K). The probe system consists of two ligand-sensing machineries: (i) a reporter-gene system carrying a glucocorticoid response element (GRE) promoter; and (ii) a single-chain probe sensing glucocorticoids. The cDNA encoding the single-chain probe is situated downstream of the GRE promoter, indicating that this system carries two on–off switches for glucocorticoids. Initially, glucocorticoids activate the GRE promoter, which starts expressing the single-chain probe. The same glucocorticoids are repeatedly recognized by the expressed single-chain probe, which emits bioluminescence. The probe system comprises both the characteristics of genetic and nongenetic probes, and thus is characteristically classified into an interface region between the two categories.

## 5. Challenges and Perspectives

Integration of multiple functions in nano-imaging agents has been witnessed in recent imaging strategies to address frontier issues in medicine and biology. This tendency appears rational because a monotonous agent exerting a singular function cannot fully reflect the complex and dynamic features of cell signaling and materials in living organisms.

This tendency of integration and complication of functions in imaging agents is observed in many probe designs; e.g., *multifunctional nanoparticles* exert fluorescence and MRI as optical signatures and can carry cargo proteins and DNAs. *Bifunctional antibodies* are developed by hybridization of a catalytic antibody with another classical antibody, thus exerting two different roles. Genetically encoded multicolor imaging probes are designed to emit two different optical signatures according to the bifacial activities of a ligand.

One of the merits of the integrative design of probes is to compensate drawbacks of individual probes. This feature is even found in the case of a representative optical signature, such as fluorescence and bioluminescence. Many of the genetically encoded fluorescent probes are unfortunately irreversible and time-consuming for chromophore formation. This fact greatly limits the quantitative analysis of temporal interactions of proteins of interest. Although the FRET methods have a strong advantage of temporal resolution, signal changes in FRET are very small so that special instrumentations with specific light filtering systems are necessary to ensure reliable sensing. The spatial resolution of fluorescent probes is generally excellent visualizing a position of individual fluorescent molecules with nanometer precision. On the other hand, the spatial resolution of genetically encoded bioluminescent probes does not reach the standard optical resolution of the fluorescent one. This is mainly because the absolute photon number of the bioluminescent systems is very low. A strong advantage of the luciferase-fragment complementation is that it enables real-time analysis of protein–protein interactions and enzyme activities, because the split fragments of luciferases are generally reversible. Therefore, it is smart to use both fluorescent and bioluminescent probes according to various experimental goals.

Future analyses with nano-imaging agents should investigate methods of non-invasive, quantitative, specific, signal-integrative, and real-time features. Their development is desired to be directed to the production of truly quantitative, highly sensitive, and easily comprehensive probes [64].

Development of excellent nano-imaging agents is greatly supported by advanced properties of light-emitting proteins and nanoparticles. Improvement in the properties of luminescent materials is directly connected to advances in molecular recognition probes. Such major advances in biological analysis can be achieved from ideas inspired by nature, similar to many of the above-introduced imaging agents that are appropriate examples of this idea. As reviewed earlier, protein splicing is a naturally occurring, self-catalytic process of premature proteins. Protein–protein complementation is one of the typical molecular events for activating signaling cascades in living cells. Likewise, we can expect advanced analytical measures in future, mimicking various molecular mechanisms of living subjects. The sense organs of the living subjects may be a prototype of the future molecular recognition probes. Furthermore, combination of the above-mentioned genetically encoded probes with nanofabricated systems would greatly advance the elucidation of complicated biological systems in living cells.

These advanced nano-imaging agents in analytical chemistry will greatly expand the scope of our knowledge, which is a common mission of analytical scientists.
